# Occupational Stress among Australia Podiatrist in general and geriatric practice

**DOI:** 10.1186/1757-1146-8-S2-P16

**Published:** 2015-09-22

**Authors:** Paul Tinley

**Affiliations:** 1 Charles Sturt University, Albury NSW 2640, Australia

**Keywords:** Occupational Stress, Podiatrist, Geriatrics

## Background

High levels of occupational stress have been reported in podiatrists practising in Australia. One possible stressor is the predominance of the treatment of aged patients with chronic disease in Podiatry practice.

## Methods

Forty (40) Podiatrists attending a regional podiatry conference were invited to participate in the research using a convenience sampling method. Podiatrists were asked to complete a survey examining occupational stress in general and specifically in relation to practice with older adults (defined as those over the age of sixty five).

## Results

The survey of sources of occupational stress among podiatrists has identified that patient demands and expectations are the most significant stressor both in general and in geriatric practice for the Podiatrist. The perceived limited clinical gains and chronic nature of their conditions in older patients was also ranked highly as a stressor.

## Conclusions

Working with the elderly is a significant part of podiatry practice and as such needs to be seen with more positive attitude by many practitioners. The development of geriatric practice as a speciality within the profession may help to raise the value of working with the elderly. This has implications for preparing podiatrists for practice with the geriatric population along with the need for strategies to avoid or minimise these work stressors.

